# Dissipation induced by phonon elastic scattering in crystals

**DOI:** 10.1038/srep34148

**Published:** 2016-09-26

**Authors:** Guolong Li, Zhongzhou Ren, Xin Zhang

**Affiliations:** 1 Department of Physics and Key Laboratory of Modern Acoustics, Nanjing University, Nanjing 210093, China; 2 Joint Center of Nuclear Science and Technology, Nanjing University, Nanjing 210093, China; 3 Center of Theoretical Nuclear Physics, National Laboratory of Heavy-Ion Accelerator, Lanzhou 730000, China

## Abstract

We demonstrate that the phonon elastic scattering leads to a dominant dissipation in crystals at low temperature. The two-level systems (TLSs) should be responsible for the elastic scattering, whereas the dissipation induced by static-point defects (SPDs) can not be neglected. One purpose of this work is to show how the energy splitting distribution of the TLS ensemble affects the dissipation. Besides, this article displays the proportion of phonon-TLS elastic scattering to total phonon dissipation. The coupling coefficient 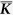 of phonon-SPD scattering and the constant *P*_0_ of the TLS distribution are important that we estimate their magnitudes in this paper. Our results is useful to understand the phonon dissipation mechanism, and give some clues to improve the performance of mechanical resonators, apply the desired defects, or reveal the atom configuration in lattice structure of disordered crystals.

In recent years, mechanical resonators have been used to excite high frequency phonons at low temperatures for investigating the quantum regime. For instance, O’Connell *et al*.^1^ realized single mechanical quantum excitation (phonon) control coupled to a qubit. Besides, the elaborate mechanical resonators can be also applied in the opto-mechanical system^2^,^3^, quantum motion^4^, gravitational wave detection^5^, and other fundamental physics^6^. However, the low quality factor (*Q*) still limits the coherence time of these quantum systems and therefore, it is crucial to manufacture high-*Q* resonators for the application^7^. In particular, Goryachev *et al*.^8^ adopted a kind of cavity resonators manufactured from the highest quality alpha-synthetic quartz, and achieved high-*Q* at frequencies from hundreds of megahertz to near 1 GHz. Therefore, these bulk acoustic wave (BAW) cavities bring a prospect to achieve operation in the equilibrium ground state of hybrid mechanical systems with longer coherence time. More interestingly, the measurement results exhibit that *Q* obeys the *Q* × *f* ^3^ = const law and tends to decrease with *T* reducing, with *f* and *T* denoting respectively the resonant frequency and the temperature. These new features manifest that there is another source limiting the quality factor of the BAW resonators made of piezo-crystals at low-temperature.

The understanding of dissipative mechanisms is of great importance for measuring, analyzing and designing mechanical resonators^9^. At high temperature that *ħω* ≪ *k*_*B*_*T*, where *ω* ≡ 2*πf* is the angular frequency of the resonant phonons, the limitation of *Q*-factor is caused mainly by the interaction of acoustic waves with thermal phonons, i.e., the anharmonic effect. At high temperature that the thermal phonon lifetime *τ* is in the *ωτ* ≪ 1 regime and even *ωτ* ~ 1 regime, the Boltzmann equation method, or Akheiser theory, can be used to calculate the dissipation from this thermal effect^10^,^11^. If *τ* is long enough at sufficiently low-temperature such that *ωτ* ≫ 1 but the thermal effect still dominates (i.e., in the *ħω* ≪ *k*_*B*_*T* regime), this anharmonic interaction can be described by phonon-phonon coupling which is regarded as a perturbation of the harmonic vibration (Landau-Rumer method)^10^,^12^,^13^. On the other hand, as temperature keeps dropping, this thermal perturbation becomes negligible and the phonon scattering by defects plays a dominant role in the dissipation. Klemens came up with a model where phonons are scattered by static-point defects (SPDs)^14^, and exhibited that the dissipation *α* is proportional to the forth power of frequency (i.e. *Q* × *f* ^3^ = const from the relation *Q* = *f*/2*α*) and independent of temperature. Except the static-point defects, the disordered parts of solids contain the ensemble of so-called two-level systems (TLSs)^15^,^16^,^17^, which has been verified experimentally^18^,^19^ and theoretically^20^. Similar with the photon-TLS interaction^21^, the TLSs in solids should be coupled to the phonon as the excitation of mechanical vibration. This kind of dynamic defect, as well as the static one mentioned above, disturbs the resonant phonons via phonon-defect coupling to cause the dissipation of mechanical resonators^22^,^23^. Instead of the resonant absorption, the phonon elastic scattering by TLSs results in a main dissipation in resonators if the energy of scattered phonons is sufficiently lower than the energy splittings of most TLSs^24^. Actually, this kind of phonon-defect scattering has been proposed before^25^, but not derived from a specific perturbation term until recent work in ref. 24. Derived from the specific phonon-TLS interaction in the condition of elastic scattering, not only does *Q* obey the *Q* × *f* ^3^ = const law, but also drops with the reduction of temperature. The model calculation is in agreement with the recent measurement result^8^.

In this paper, we further discuss the phonon-TLS scattering mechanism under consideration of the PSD scattering mentioned above, and obtain some quantitative results in comparison with the recent experiment^8^. In other words, this work is beyond the theoretical framework mentioned in ref. 24 that just discussed the effect from TLS without the static-point defeat, and relates with experiments that should be influenced by both effects. First, we show that the distribution of energy splitting of TLSs influences the *Q* dependence on temperature. It provides a method to infer the distribution of energy splitting of TLS ensemble via measuring the *Q*-factor at various temperature. Besides, now that the PSD contribution is taken into account, we also estimate the relative contribution from phonon-TLS scattering at several temperature after determining the distribution of energy splitting of TLSs. In the end, two parameters, including the coupling coefficient 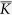 of phonon-SPD scattering and the constant *P*_0_ of the TLS distribution, that are crucial for yielding phonon dissipation also need to be estimated and discussed. Our results are available for improving and testing the phonon dissipation mechanism, and are helpful to improve the performance of mechanical resonators, apply the desired defects, or reveal the atom configuration in lattice structure of disordered crystals.

## Results

### The models

For sound wave propagating in crystals, the mechanical harmonic vibration breaks since the microscopic impurities randomly distribute in crystals^26^. The details for quantizing mechanical waves are given in Methods. These static-point defects lead to phonon elastic scattering for two reasons, including (a) the different mass between lattice atoms and impurities, and (b) the different binding to neighbors between of lattice atoms and of impurities. This perturbation Hamiltonian *H*_*i*_ is described as^27^


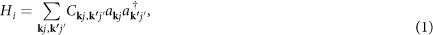


where *a*_**k***j*_ (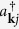) is the annihilation (creation) operator of the mechanical mode with wave-vector **k** and polarization *j*, corresponding to the normal-mode frequency *ω*_**k***j*_. The polarizations contain *j* = *l*, *t* for a longitudinal and two transverse branches, depending on whether the polarization vector **e**(**k***j*) is parallel or perpendicular to the wave-vector **k**. In the case of long-wavelength phonons with the linear dispersive relation, the coefficient *C*_**k***j*,**k**′*j*′_ for mode (**k**, *j*) scattered elastically to (**k**′, *j*′) is given by^14^


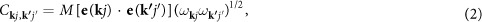


where *M* describes the change or the binding-force changes at impurity position **r** in crystals.

**Figure 1:**
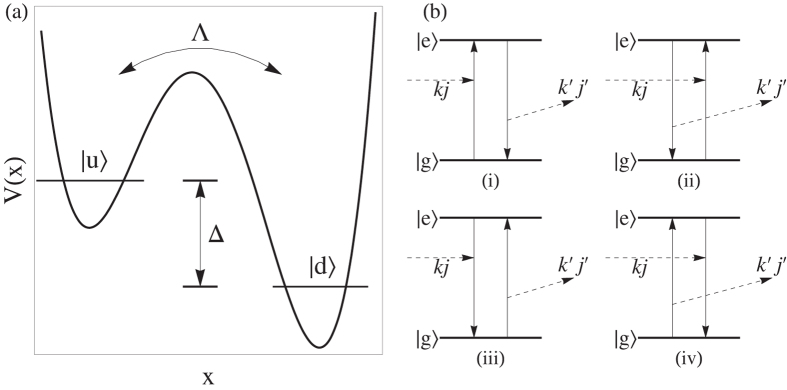
(**a**) A TLS including two stable states. Δ and Λ denote the separation and the tunneling between the upper state |*u*〉 and the lower one |*d*〉, respectively. (**b**) Four elastic scattering processes due to the TLSs. Each dashed arrow with *kj* indicates incident or released phonon with wave vector **k** and polarization *j*.

According to the previous works^28^,^29^,^30^, two-level systems (TLSs) have been suggested to exist in disordered part of crystals. Two parameters are needed to describe a TLS: the asymmetry Δ and the tunneling Λ between the two bound states (see Fig. 1(a)). The effective Hamiltonian of a TLS can be written as


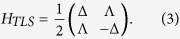


This is equivalent to a TLS with an energy splitting 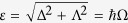 (the angular transition frequency Ω = 2*πν*) between the eigen ground state |*g*〉 and exited state |*e*〉. On the other hand, the coupling between strain field and a TLS happens mainly through the change of the asymmetry *δ*^31^,^32^. Therefore, the perturbation Hamiltonian for the coupling is given by


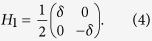


Here, *δ* is linear in the strain tensor field, *δ* = 2*γ*_*ij*_*S*_*ij*_, where *S*_*ij*_ = (∂_*i*_*u*_*j*_ + ∂_*j*_*u*_*i*_)/2 expresses the strain tensor with the displacement field (16), and *γ*_*ij*_ indicates the linear coefficient. Once the Hamiltonian (3) is diagonalized^17^,^33^, we can obtain this phonon-TLS coupling in terms of phonon creation and annihilation operators,


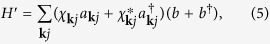


with the coefficient 
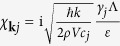
 and the rasing and lowing operators for TLSs, i.e., *b*^ + ^ and *b*. Here, *ρ* and *V* denote respectively the mass density and volume. In the phonon-TLS interaction (5), we only consider the longitudinal and transverse values of *γ*_*ij*_, denoted as *γ*_*j*_ with polarization *j* = *l* or *t*.

The phonon-TLS interaction leads to phonon elastic scattering, and four processes make contributions to the scattering amplitude *A*_**k**′*j*′,**k***j*_ from mode (**k**, *j*) into mode (**k**′, *j*′) (schematic diagram can be seen in Fig. 1(b) and calculation details in Methods). On the basis of second-order perturbation theory^34^, the amplitude *A*_**k***j*,**k**′*j*′_ is equivalent to an effective Hamiltonian as


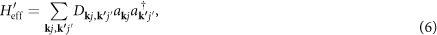


with coefficient


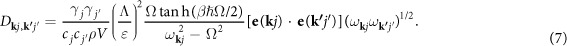


For the small value of *ω*_**k***j*_/Ω, this coefficient can be written approximately as


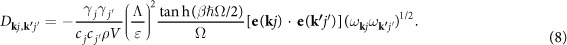


The *Q* ~ *ω*^−3^ law. Applying fermi’s golden rule with equation (1), we can derive the dissipation of a phonon with frequency *ω* undergoing elastic scattering by static-point defects,


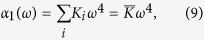


where we have considered the sum of factors *K*_*i*_ of various impurity contributions, denoted by factor 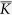. Nevertheless, the temperature independence of equation (9) means that this mechanism can not explain fully the measured results in ref. 8.

On the other hand, the dynamic defeats, i.e., TLS, should be taken into account. We obtain this dissipation of a longitudinal phonon (since the shear modes have larger dissipations and thus are difficult to couple to higher frequencies) scattered elastically by TLSs based on the Hamiltonian (6),


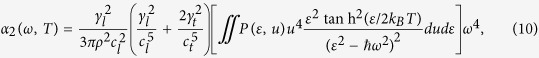


with parameter *u* = Λ/*ε*. We have introduced the function *VP*(*ε*, *u*) as the distribution density of parameters *ε* and *u*, and thus the total dissipation *α* is expressed by an integration if these parameters of the TLS ensemble are regarded as a continuous distribution. In standard tunneling model^35^,^36^, the distribution function has the form 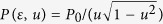, with the constant *P*_0_. Hence, the formula (10) becomes


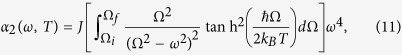


with the coefficient 
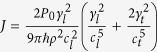
 and the lower and bounds Ω_*i*,*j*_ = 2*πν*_*i*,*j*_. If Ω_*i*_ is much larger than the resonant frequency *ω*, the attenuation becomes


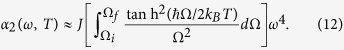


It means that, if TLSs with high energy splitting dominate in crystals, most of excited phonons undergo elastic scattering by TLS ensemble and their dissipation formula (12) derived from phonon-TLS interaction (5) is proportional approximately to the fourth power of frequency. But unlike the static scattering mechanism, the dissipation resulted from TLSs drops with temperature rising. The physical interpretation for this anomalous *T*-dependence is that, at lower temperature, more TLSs are in their ground states to absorb resonant phonons and re-emit them in a random direction, leading to phonon dissipation. However, this anomalous temperature trend can not last at a higher temperature where phonon-phonon scattering prevails over the elastic scattering.

Finally, both independent dissipative mechanisms should be considered together, and then based on equations (9) and (11) the total quality factor *Q* is expressed as


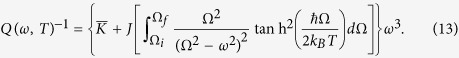


According to equations (8) or (12) in the condition *ω* ≪ Ω_*i*_, the above formula becomes


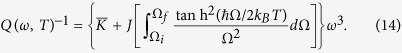


**Figure 2:**
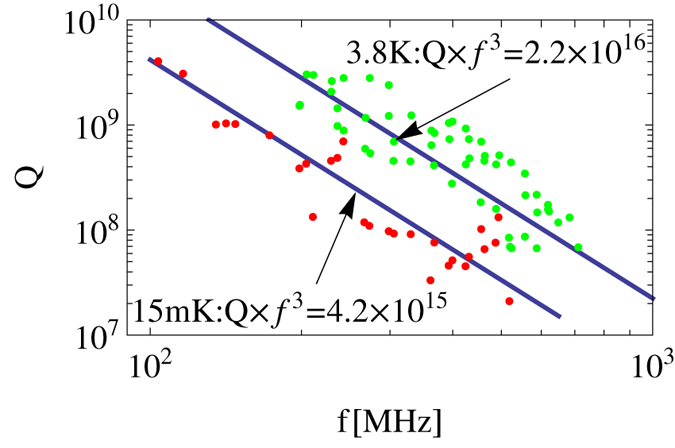
The comparison between our formula (13) and the experimental data^8^, which are denoted by solid lines and points, respectively.

Obviously, equation (14) follows the *Q* × *f* ^3^ = const law at a given temperature, and due to the second term in curly braces, quality factor rises as temperature increases. This result is in agreement with the recent experiment^8^. Just as shown in Fig. 2, the measurement data obey the *f*^−3^ law, with const = 2.2 × 10^16^ (4.2 × 10^15^) [MHz]^3^ at 3.8 K (15 mK).

### Temperature dependence influenced by the distribution of TLSs

As shown in Fig. 2, the constant of the product *Q* ⋅ *f* ^3^  changes with temperature. Back the formula (13), the term that is responsible for the influence of temperature also depends on the distribution of energy splitting of TLSs. However, this distribution has not been obtained from experiments yet, and we can therefore analyze how the range of energy splitting of the TLS ensemble affects the scattering rate so far.

**Figure 3:**
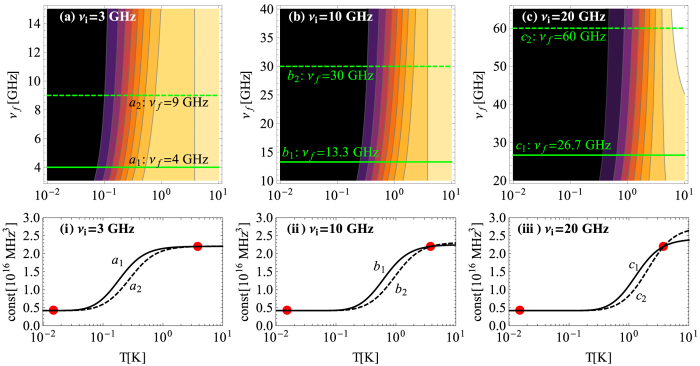
The dependence of the constant for the product *Q* · *f* ^3^  on temperature, influenced by the range of frequency splitting. The upper panels (**a–c**) indicate the situations for the lower bound *ν*_*i*_ = 2 GHz, 10 GHz and 20 GHz, respectively. The lower panels (i)–(iii) respectively correspond to the upper panels (**a–c**), and each of them contains two lines for displaying the temperature dependences at two kinds of upper bound *ν*_*f*_ indicated via two lines in each of upper panels (**a–c**). Besides, the circle points in the lower panels are fixed due to the experiment^8^, i.e., const = 4.2 × 10^15^ at 15 mK and 2.2 × 10^16^ at 3.8 K in Fig. 2.

It is necessary to take the parameters for formula (14), including both longitudinal and transverse coupling parameters *γ*_*l*_ = 0.6 eV and *γ*_*t*_ = 0.4 eV, both longitudinal and transverse sound speeds *c*_*l*_ ≈ 7 × 10^3^ m/s and *c*_*t*_ ≈ 4 × 10^3^ m/s^7^, and the mass density of quartz *ρ* = 2.6 × 10^3^ kg/m^3^ 37. In addition, the undetermined constant of the *u*-distribution *P*_0_ is discussed below. In Fig. 3, we display the product constant of *Q* ⋅ *f* ^3^  as a function of temperature from 0.01 to 10 K with different ranges of transition frequency of the TLS ensemble. We take *ν*_*i*_ = 3 GHz, 10 GHz and 20 GHz corresponding respectively to the upper panels (a–c). The lower panels (i–iii) are chosen by the lines in panels (a–c), respectively. Besides, the curves in three lower panels are fixed partly by two measured points exhibited in Fig. 2. All panels in this figure show that the constant and thus the quality factor turn higher with temperature rising, in the situation where thermal-induced dissipation is still restricted at such low temperature.

For low *ν*_*i*_ [at least *ν*_*i*_ ≤ 10 GHz, see Fig. 3(a,b)], the constant with highly narrow-*ν* range increases with temperature and maintains larger than the one with relatively wide-*ν* range. Nevertheless, whether the *ν* range is narrow or wide, the temperature trend stops rising and the magnitude of the constant turns to a same value at several Kevin [see Fig. 3(i,ii)]. Besides, as the *ν*_*f*_ is up to high enough, the temperature trend nearly exhibits same behaviour even if the *ν* range is wider. In other words, the magnitude of *ν*_*i*_ plays a decisive role in the constant for the wide *ν* range. On the other hand, in high *ν*_*i*_ regime [see Fig. 3(c)], the constant with highly narrow-*ν* range still rises with temperature and keeps larger than the one with relatively wide-*ν* range in the temperature region of near 1 K, whereas the the latter exceeds the former at several Kelvin [see Fig. 3(iii)]. It seems that the TLS ensemble with wider transition frequency spectrum leads to more rapid increase of the constant with temperature rising at several Kelvin. In the relatively low temperature region of 10^−2^ −10^−1^ K, the lines of panels (ii) and (iii) in Fig. 3 hardly change with keeping a stable value. Compared with these cases of high *ν*_*i*_, the panel (i) displays that the constant starts rising in this low temperature region. Apart from this, the comparison between panels (ii) and (iii) also shows this feature that the stable situation is in a wider range in low temperature region for higher *ν*_*i*_ situation.

The above analysis provides a clue for future experiments to reveal the distribution of energy splittings of TLS ensemble in crystals. In future measurements for *Q*-factor, if the line of the frequency behaviour of *Q* at *T* > 3.8 K is higher than the one at *T* = 3.8 K in Fig. 2, it will mean that the energy splittings of most TLSs lie in higher energy range. When the difference between these two lines is larger, the transition frequencies have a wider range from lower limit *ν*_*i*_ to upper limit *ν*_*f*_. On the contrary, if the line at higher temperature is close to the one at 3.8 K, it is reasonable to infer that there are considerable TLSs with low energy splittings. In this situation, the *Q*-factor at medium temperature should be measured to determine the energy splitting range of the TLS ensemble, based on the temperature behaviour in Fig. 3(a,b) and (i,ii). However, once the range is wide enough, the *ν*_*f*_ can not be determined by this means, but the only decisive value *ν*_*i*_.

### The contribution from phonon-TLS scattering *R*_TLS_

Now that both TLSs and PSDs participate in the phonon elastic scattering, it is necessary to analyze and discuss their proportion to the phonon dissipation. Even though the parameters 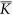 and *P*_0_ are unknown, the relative contribution of phonon-TLS elastic scattering can be estimated via comparing the formula (14) with the experiment in ref. 8. In this paper, we take the ratio of the inverse of *Q* ⋅ *f* ^3^  product only considering the TLS contribution to the total one including the both contributions of these scattering mechanisms mentioned above, denoted by *R*_TLS_, as the relative contribution of phonon-TLS scattering.

**Figure 4:**
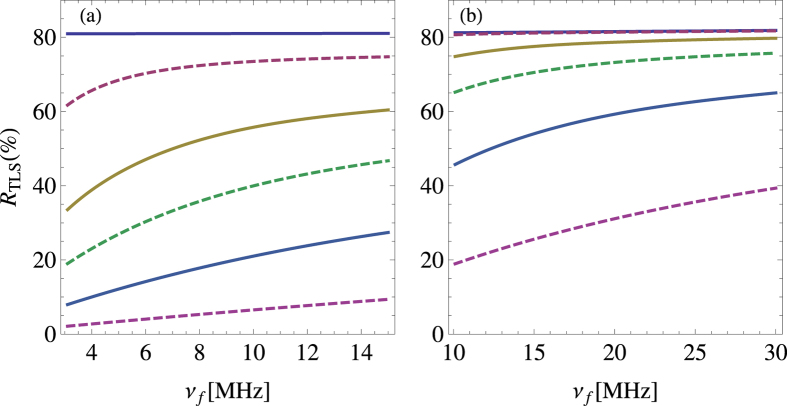
The relative contribution from phonon-TLS scattering, R_TLS_, for various ranges of transition frequency *ν* of TLS ensemble. (**a,b**) Depict the situation for *ν*_*i*_ = 3 GHz and 10 GHz, respectively. In each figure, the lines from upper to lower indicate the various trends at *T* = 0.01, 0.1, 0.2, 0.3, 0.5 and 1 K, respectively. In particular, the lines for *T* = 0.01 and 0.1 K nearly overlap each other in panel (b).

As illustrated in Fig. 4, its panels (a) and (b) depict the situation for fixing *ν*_*i*_ = 3 GHz and 10 GHz, respectively. Whether the TLS ensemble lies in low- or high-transition frequency region, the relative contribution *R*_TLS_ is higher at lower temperature. Especially at dozens of milli-kelvin, the phonon-TLS elastic scattering is the dominative dissipation mechanism. Just because of the different ratios at various temperature, the experiment in ref. 8 displays the temperature dependence of elastic scattering rate which results from phonon-TLS scattering. Additionally, all lines at different temperature rises with *ν*_*f*_ increasing. The line at 0.1 K is close to the one at 0.01 K in the Fig. 4(b), in comparison with the two lines in the Fig. 4(a). At several Kelvin, while it seems that the TLS ensemble hardly takes part in the dissipation processes in the Fig. 4(a), the Fig. 4(b) shows that the contribution from the TLS ensemble is considerable. This difference of these two panels manifests that the TLSs with high transition frequency lead to the major contribution of phonon-TLS scattering at a given temperature.

### The coupling strength of phonon-SPD scattering 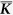


**Figure 5:**
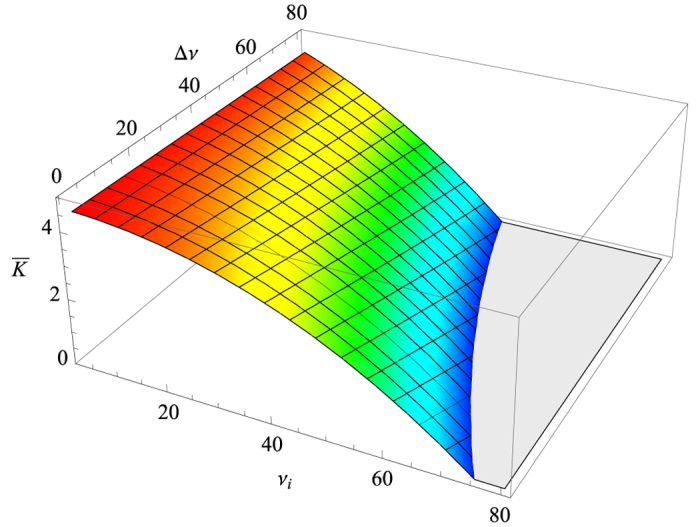
The phonon-PSD coupling coefficient 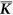 as a function of the lower bound *ν*_*i*_ and the range Δ*ν* = *ν*_*f*_ − *ν*_*i*_. The unit of 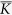 is 10^−17^ MHz^−3^, while the units of *ν*_*i*_ and Δ*ν* are both GHz. The color indicates the magnitude of 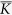. There is an unallowed region of the *ν*_*i*_ − Δ*ν* plane, otherwise the coefficient 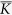 enters the negative region.

The above analysis reveals that quality factor at a given temperature is determined by the distribution of energy splitting of TLSs. Apart from the TLS scattering which is responsible for the temperature dependence, the static-point defects also make a contribution to the phonon elastic scattering. The coupling strength 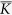 of this kind scattering can be determined at fixed lower bound *ν*_*i*_ and range Δ*ν* = *ν*_*f*_ − *ν*_*i*_, according to formula (13) and the parameters given above, as shown in Fig. 5.

In Fig. 5, the elastic scattering can not occur in the colorless region, otherwise the coefficient 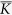 enters the unallowed negative region. As a consequence, it manifests that, at a specific range Δ*ν*, the lower bound *ν*_*i*_ has a maximum magnitude that decreases with Δ*ν* increasing. Besides, if the lowest transition frequency *ν*_*i*_ is about several gigahertz, the coefficient 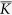 is nearly unchanged no matter if the range Δ*ν* is extremely narrow or wide as dozens of gigahertz. However, the stable state of 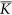 can not last as long as *ν*_*i*_ exceeds the several gigahertz region, which makes an obvious difference between the narrow and wide ranges Δ*ν* at a given high *ν*_*i*_. On the other hand, the coefficient 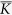 turns out to be more sensitive to *ν*_*i*_ and decreases to zero more rapidly if the range Δ*ν* is wider, also leading to the obvious difference of 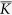 for various Δ*ν* in high-*ν*_*i*_ regime. In a conclusion, the distribution of the transition frequencies that responds to the largest 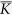 concentrates narrowly on low *ν*_*i*_ region. In other words, the TLSs with higher transition frequency result more possibly in their scattering with phonons, and thus the contribution from SPD scattering becomes less as well as coupling coefficient 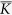.

### The distribution constant *P*_0_

Just as the coupling coefficient 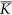 mentioned in above section, the constant *P*_0_ is also crucial for obtaining the model results and can be determined on the basis of the experiment in ref. 8 once *ν*_*i*_ and Δ*ν* are measured.

**Figure 6:**
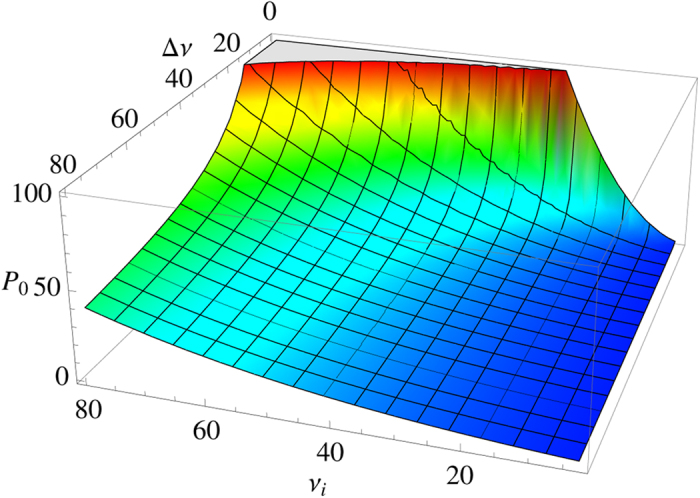
The distribution constant *P*_0_ as a function of *ν*_*i*_ and Δ*ν*. The unit of *P*_0_ is 10^50^ J^−1^ m^−3^, while the units of *ν*_*i*_ and Δ*ν* are both GHz. The color indicates the magnitude of *P*_0_.

The Fig. 6 plots the magnitude of constant *P*_0_ and its dependence on both *ν*_*i*_ and Δ*ν*. If *ν*_*i*_ is low with narrow or wide range Δ*ν*, *P*_0_ is turned out to be suppressed highly, especially in wide range case. Despite all this, *P*_0_ still gets large with *ν*_*i*_ rising, and this increase is highly rapid for extremely narrow spectrums of transition frequency *ν*. On the other hand, as the density of the transition frequency, the constant *P*_0_ also tends to be larger in the case that the spectrum of transition frequency *ν* is narrower at a given *ν*_*i*_, and this parameter increases more steeply as *ν*_*i*_ enter higher region. In general, the magnitude of *P*_0_ increases rapidly as the transition frequencies of the TLS ensemble turn to highly concentrate in high regime.

## Discussion

In this paper, we first combine both the mechanisms of phonon dissipation, including the elastic scattering with two-level systems (TLSs) and point-static defects (PSDs), to obtain the quality factor (*Q*) formula (13) at low temperature. We then find that *Q* follows the *Q* ⋅ *f* ^3^ = const law, expressed by equation (14), in bulk mechanical resonators made of piezoelectric quartz. The phonon-TLS scattering reveals that the *Q*-factor rises with *T* increasing until, at a higher temperature, the anharmonic effect plays a considerable role in the mechanical dissipation. These frequency and temperature features of quality factor conform to the recent cryogenic measurement in ref. 8 (see Fig. 2). In conclusion, this work improves the theoretical framework in ref. 24 with full consideration (containing both static and dynamic defeats), and provides direct comparison with experiment and more quantitative analysis.

The detail of this paper is our quantitative results on the basis of the theoretical model. First of all, the distribution of energy splitting of the TLS ensemble should be considered to obtain the explicit *T*-dependency (see Fig. 3). These results are obtained via combining our formula equation (14) with the measurement in ref. 8. If there are considerable TLSs having low transition frequency *ν* (i.e. several gigahertz), the constant of *Q* ⋅ *f* ^3^  product, as well as *Q*, rises from low to high temperature with larger magnitude for the narrower *ν* spectrum. When the temperature is up to several Kelvin, the constant stops increasing and keeps a stable value. On the contrary, if transition frequencies of the TLS ensemble concentrate in higher regime (i.e. dozens of gigahertz), the temperature curve goes up more steeply and keeps this increase at several Kelvin. This situation becomes more obvious as the quartz crystal contains more TLSs with higher *ν*. This behaviour can be applied to infer the distribution of the transition frequency of the TLS ensemble. Once the distribution of the transition frequency is determined via experiments, the relative contribution of the phonon-TLS scattering to the total scattering rate can be estimated (see Fig. 4). As the temperature is lower, this relative contribution *R*_TLS_ is larger and thus the phonon-TLS scattering is more dominant for the phonon dissipation. At a given temperature, the higher *ν*_*i*_ leads to larger proportion of the phonon-TLS scattering which still continues rising with *ν*_*f*_ increasing. It manifests that the TLSs with high transition frequency lead to the major contribution of phonon-TLS scattering at a given temperature. Furthermore, now that we have included the phonon elastic scattering induced by static-point defects, its effective coupling coefficient 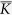 can be estimated from the *Q* formulas (13) and (14) via comparing with the experimental results (see Fig. 5). The distribution of the transition frequencies that responds to the largest 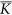 concentrates narrowly on low *ν*_*i*_ region. At last, it is also important to estimate the contribution constant *P*_0_ for the final dissipation, and the Fig. 6 illustrates the magnitude of constant *P*_0_ and its dependence on both *ν*_*i*_ and Δ*ν*. In general, the value of *P*_0_ increases rapidly as the transition frequencies of the TLS ensemble turn to highly concentrate in high regime.

The model describes the phonon dissipation, as well as *Q*, at low temperature due to the existence of various defects in crystals. Based on our results, further quantum experiments which are coupled with the mechanical systems with TLS defects^1^,^18^,^38^ can test whether our theory fits the physical facts. Moreover, we believe that our results give some clues to improve the performance of mechanical resonators^7^,^8^,^39^,^40^, apply the desired defects^41^, or reveal the atom configuration in lattice structure of disordered crystals.

## Methods

### The quantized method for mechanical waves

Our calculations are on basis of the quantization of the sound wave propagating in crystals. The quantized noninteracting phonon Hamiltonian of an ideal harmonic crystal can be expressed as


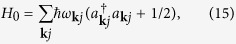


where *a*_**k***j*_ (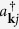) is the annihilation (creation) operator of the mechanical mode with wave-vector **k** and polarization *j*, corresponding to the normal-mode frequency *ω*_**k***j*_. The theory is quantized via the standard commutation relation 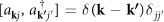. The quantized displacement field at position **r** is then written as a sum of traveling waves,


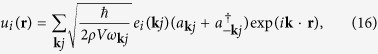


where *e*_*i*_(**k***j*) is the *i*-component of the unit polarization vector **e**(**k***j*) for given **k** and *j*, and *ρ* and *V* indicate respectively the mass density and the volume of crystals. For long-wavelength acoustic wave, the dispersion relation can be expressed linearly as *ω*_**k***j*_ = *c*_*j*_*k*, where *c*_*j*_ is the sound velocity with polarization *j* in solids and *k* = |**k**|.

### The perturbation method for obtaining equation (6)

Let *A*_**k***j*,**k**′*j*′_ denote the total amplitude of phonon elastic scattering by a TLS with energy splitting *ε* ≡ *ħ*Ω. On the basis of the perturbation term (5) and the second-order perturbation theory^34^, the contributions of processes (i)–(iv) in Fig. 1(b) to the total amplitude *A*_**k***j*,**k**′*j*′_ are respectively as following:


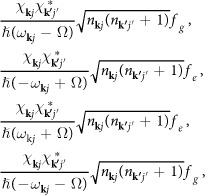


where *n*_**k***j*_ denotes the phonon population of mode (**k**, *j*), and the factors *f*_*g*_ and *f*_*e*_ are as the probability for TLSs in ground and excited states, respectively. Obviously, The sum of above four formulae is the total amplitude


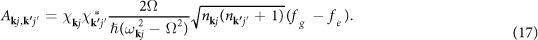


Take *f*_*g*_ − *f*_*e*_ = tan h(*βħ*Ω/2) for thermal equilibrium atoms in solid.

### Fermi’s golden rule of perturbation method

According to Fermi’s golden rule of perturbation theory^14^, the attenuation for mode (**k**, *j*) is written as


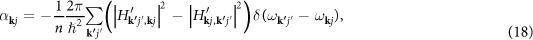


where *H*′ is the perturbation term of Hamiltonian, i.e., the equations (1) and (6) in this paper, and both 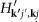 and 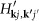 indicate the matrix elements 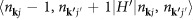 and 〈*n*_**k***j*_ + 1, *n*_**k**′*j*′_ − 1|*H*′|*n*_**k***j*_, *n*_**k**′*j*′_〉 respectively. Besides, *n* is defined as deviation from equilibrium for mode (**k**, *j*) in the above equation, i.e., 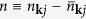 with the Bose-Einstein distribution 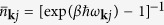. Then taking the perturbation terms (1) and (6), the attenuation formulae (9) and (10) can be obtained respectively.

## Additional Information

**How to cite this article**: Li, G. *et al*. Dissipation induced by phonon elastic scattering in crystals. *Sci. Rep.* **6**, 34148; doi: 10.1038/srep34148 (2016).
